# Comparative Short-Term Outcomes of Conservative Management Versus External Ventricular Drainage in Adults With Spontaneous Intraventricular Hemorrhage: A Mixed Observational Cohort Study

**DOI:** 10.7759/cureus.101220

**Published:** 2026-01-10

**Authors:** Mohamed Desoky, Mostafa Mubarez, Mohamed Soliman, Mohamed Melegy

**Affiliations:** 1 Neurosurgery, Faculty of Medicine, Al-Azhar University, Assiut, EGY; 2 Neurosurgery, Faculty of Medicine, Al-Azhar University, Cairo, EGY; 3 Neurosurgery, Faculty of Medicine for Girls, Al-Azhar University, Cairo, EGY

**Keywords:** conservative treatment, early surgical intervention, external ventricular drainage, glasgow coma scale, intracerebral hemorrhage, primary intraventricular hemorrhage

## Abstract

Background: Spontaneous intraventricular hemorrhage (IVH) is a rare and serious condition with high morbidity and mortality, often leading to hydrocephalus and elevated intracranial pressure. Optimal management, surgical versus conservative, remains a topic of debate, particularly when hydrocephalus is present.

Objective: This study aimed to compare clinical outcomes in adult patients with spontaneous IVH managed conservatively versus those undergoing external ventricular drainage (EVD).

Patients and methods: This single-center mixed retrospective-prospective observational study included 40 adult patients with spontaneous IVH managed either conservatively or with EVD. All patients underwent full clinical assessment, brain magnetic resonance imaging (MRI), and computed tomography (CT) angiography. Outcome was measured using Glasgow Coma Scale (GCS) changes at discharge. Comparisons between groups were conducted using chi-squared tests for categorical variables and t-tests or Mann-Whitney U tests for continuous variables.

Results: There were insignificant variances in sex, age, Graeb score, or follow-up CT findings between the two groups. However, hemiparesis was significantly more frequent in the surgical group (85% vs. 55%; P=0.038). Conservative treatment resulted in a significantly higher improvement rate (90% against 60%; P=0.028) and lower mortality (10% vs. 40%).

Conclusion: Conservative treatment in selected adult patients with spontaneous IVH was associated with better short-term outcomes in terms of functional recovery and survival compared to surgical interventions, which were associated with more neurological complications and higher mortality. However, these findings should be interpreted in light of baseline severity differences that may have influenced treatment selection.

## Introduction

Spontaneous intraventricular hemorrhage (IVH) confined to the ventricular system is a rare but clinically important entity, representing approximately 3% of all spontaneous intracranial hemorrhages [1]. IVH is associated with a high likelihood of acute obstructive hydrocephalus, followed by prompt neurological deterioration and death if not treated promptly. External ventricular drainage (EVD) is typically required for immediate treatment, but even with intervention, short-term mortality is elevated, with registry data showing 30-day mortality of almost 29% and good functional recovery in only a third of patients [2].

IVH is most frequently a complication of intracerebral hemorrhage (ICH) or subarachnoid hemorrhage (SAH) but may appear as an isolated primary event [3]. Blood products found in the ventricular system inhibit cerebrospinal fluid (CSF) flow, leading to acute hydrocephalus and increased intracranial pressure. Hemoglobin breakdown products lead to inflammatory and cytotoxic damage to the periventricular structures (e.g., hypothalamus, brainstem) beyond an obstructive effect, as shown with experimental studies and neuropathological evidence, further extending neurological injury and poor outcome [4,5].

The etiology of primary IVH remains heterogeneous. The most frequently encountered causes are vascular malformations, such as arteriovenous malformation (AVM), cavernous malformations, and, less commonly, aneurysms, which can underlie primary IVH; nonetheless, many cases remain lesion-negative [6]. However, in as many as half of the patients, no structural lesion is detected on careful examination. Chronic hypertension has been implicated as a probable cause, possibly by way of occult microhemorrhages in the periventricular regions that extend directly into the ventricular system [7]. Such mechanisms emphasize the challenge of making a differential diagnosis between true primary IVH and secondary extension from parenchymal hemorrhages.

Primary IVH patients typically present with acute headache, nausea, vomiting, and varying levels of altered consciousness, ranging from disorientation to coma [8]. Focal neurological deficits are less common but may occur in the context of hydrocephalus or brainstem compression, though false-localizing cranial nerve palsies, particularly third and sixth nerve palsies, can happen due to brainstem distortion [9]. The Glasgow Coma Scale (GCS) remains a potent prognostic factor, whereby admission scores of low values correlate strongly with mortality and poor functional outcome. Of importance, patients are also at risk of abrupt deterioration from recurring bleeding, hydrocephalus, or other complications and thus need close clinical monitoring [10].

Management of IVH is multifaceted and is directed to the control of hemorrhage, relief of hydrocephalus, and stabilization of intracranial pressure. While ultimate management of the underlying etiology such as the obliteration of an aneurysm or treatment of AVM is important, EVD remains the cornerstone for cases complicated by acute hydrocephalus or raised intracranial pressure [11]. However, it is marred by catheter occlusion, ventriculitis, and shunt dependency, with complication rates in some series exceeding 35% [2]. New technologies like neuroendoscopic brainwashing and active CSF irrigation have had superior short- and long-term outcomes compared to EVD alone [12,13]. Even with improvements in neurocritical care, there is still substantial uncertainty about the best management approach for spontaneous IVH, specifically regarding the relative benefit of treatment with and without intervention versus EVD placement. Current clinical practice is heterogeneous across institutions, and we do not have a consensus about which subgroup of patients (if any) has demonstrated to date anything but minor benefit in relation to EVD placement. This lack of knowledge will require targeted comparative studies to measure short-term outcomes in usual clinical practice.

This study thus aims to compare and assess different management modalities for spontaneous IVH in adults, including primary IVH and IVH secondary to ICH or SAH.

## Materials and methods

Study design and characteristics of participants

This study utilized a single-center mixed retrospective-prospective observational cohort and was conducted at Al-Azhar Assiut University Hospital, Assiut, Egypt. The retrospective group included patients with spontaneous IVH admitted and treated between February 2023 and January 2024. The prospective group included newly admitted patients who were enrolled and followed between February 2025 and June 2025 after receiving institutional review board (IRB) approval. Both cohorts followed the same institutional protocol for triage, imaging, EVD indications, and outcome assessment and were evaluated utilizing the same inclusion and exclusion criteria and standardized clinical and imaging processes to maintain consistency throughout the study period. Treatment strategy (conservative vs. EVD) was chosen by the clinical team, which made two study groups: group A consisted of 20 patients who were treated conservatively, whereas group B consisted of 20 patients who underwent surgical management. 

Patient recruitment

This study involved adult patients (age ≥15) with spontaneous IVH, including primary IVH and secondary IVH due to ICH, aneurysmal SAH, or AVM. Lesion intervention (coiling, clipping, or AVM resection) was documented.

Exclusion criteria included post-traumatic IVH, anticoagulation hemorrhage without reversal, tumor-related bleeds or mycotic aneurysms, pregnancy, extensive shunt procedures, and patients without an available baseline computed tomography (CT).

Data collection and clinical workup

All patients underwent thorough history taking, clinical examination, and initial investigations. Patients underwent neuroimaging, a combination of magnetic resonance imaging (MRI) and CT cerebral angiography. The MRI studies included T1-weighted, T2-weighted, fluid-attenuated inversion recovery (FLAIR), diffusion-weighted imaging (DWI), susceptibility-weighted imaging (SWI), and post-contrast T1 sequences as appropriate. These sequences were used to examine for subtle parenchymal or IVH, evaluate for a vascular or neoplastic lesion, and rule out other secondary hemorrhagic causes, particularly in the posterior fossa, where CT may be limited by artifacts. Patients were scanned in a supine position using a dedicated head coil, and all imaging was conducted using standard brain sequences while the technologist remained continuously present.

CT cerebral angiography was conducted in a supine position, starting with a scout image from the mid-chest to the cranial vertex and proceeding with caudocranial scanning from the aortic arch to the vertex. A non-ionic iodinated contrast agent (50-75 ml), followed by a 100 ml saline chaser at a rate of 4.5-5 ml/s, was injected via automated contrast injection. Breath-hold techniques were utilized to prevent motion artifacts, and the contrast threshold for the augmentation of the vascular circulation was 100 Hounsfield units (HU). 

Surgical management

Surgical management consisted of EVD, which was completed in the operating room under sterile conditions. We placed EVDs in individuals with altered consciousness due to acute obstructive hydrocephalus, radiological ventricular enlargement, or signs of intracranial hypertension. Timing of insertion relative to arrival at the emergency department was recorded (median 2-4 hours).

The ventricular catheter was placed typically within the frontal horn with the side of insertion at the discretion of the operating neurosurgeon. The EVD chamber was most often set to 5 cm H₂O. All patients received prophylactic intravenous ceftriaxone one hour prior to the EVD and continued during the time of drainage as well as for continued care. CSF samples were extracted from the drainage collection every 48 hours for microbiology cultures to monitor for infection.

Typically, these drains were used for 3-7 days. During weaning, the height of the drainage was progressively elevated, and clamping trials were also performed. Our institution did not give routine intraventricular thrombolytics during the study period. EVD complications were prospectively recorded: catheter occlusion requiring catheter reposition, ventriculitis as defined by the Centers for Disease Control and Prevention/National Healthcare Safety Network (CDC/NHSN), and need for permanent CSF diversion (ventriculoperitoneal shunt) before discharge.

Conservative management

Conservative management was initiated to include blood pressure management, hyperosmolar agents, intravenous fluids, H₂ receptor antagonists, anticonvulsants, early nutritional support, and initiation of physical therapy. Conservative management was designed to ensure the clinical stabilization of all patients in addition to the prevention of secondary neurological deterioration during recovery.

Outcome measures and follow-up

The main endpoint was in-hospital mortality. Secondary endpoints included short-term clinical improvement, defined as an increase of ≥2 points on the GCS measured at hospital discharge, and radiographic evolution on follow-up images. A discharge GCS was used as a pragmatic short-term measure because we had limited long-term follow-up, particularly in the retrospective cohort. Functional scales, such as the modified Rankin Scale (mRS) or the Glasgow Outcome Scale (GOS) at 30-90 days, were not uniformly documented and thus could not be analyzed.

Statistical analysis

Data were analyzed using jamovi (Version 26) ([Computer Software]. Retrieved from https://www.jamovi.org). Continuous variables were expressed as mean±SD and categorical variables as frequencies and percentages. Student's t-test or Mann-Whitney U test was applied for continuous data and the chi-squared test for categorical variables. A p-value of <0.05 was considered statistically significant.

Ethical considerations

The study protocol was approved by the Research Ethics Committee of the Faculty of Medicine for Girls, Al-Azhar University (FMG-IRB) (approval number: 2539; date: 15-10-2024). Written informed consent was obtained from all participants or their legal guardians for the prospective part of the study. Research participants' confidentiality and data privacy were protected during study conduct consistent with institutional and international ethical guidelines.

## Results

Baseline and clinical characteristics of the study participants

Forty patients presented with spontaneous IVH. The median age for this cohort was 49.3±11.5 years, with 58% (n=23) being males. Admission GCS scores averaged 11.7±1.94. The underlying etiology comprised ICH in 65% (n=26), SAH in 25% (n=10), and AVM in 10% (n=4).

Hypertension was present in 53% of patients (n=21) and diabetes mellitus in 20% (n=8). Cardiac risk factors were documented in 28% of the cohort (n=11), including atrial fibrillation (10%; n=4), ischemic heart disease (7.5%; n=3), combined IHD with valvular disease (5%; n=2), and heart failure (5%; n=2). Renal complications were identified in 10% (n=4): chronic kidney disease (50%; n=2), renal artery aneurysm (25%; n=1), and renal artery stenosis (25%; n=1). Respiratory risk factors affected 7.5% (n=3), with chronic obstructive pulmonary disease in 67% (n=2) and aspiration pneumonia in 33% (n=1).

The burden of IVH on admission, stratified by the Graeb score, included mild disease (33%; n=13) with a score of 1-4, moderate disease (48%; n=19) with a score of 5-8, and severe disease (20%; n=8) with a score of 9-12. Hemiparesis was present in 73% of the cohort (n=29) and absent in 11 (28%) patients (Table 1).

**Table 1:** Baseline demographic and clinical characteristics of the study cohort (n=40) ¹Continuous data are presented as mean and standard deviation or n (%). Percentages within each risk factor category are expressed relative to the number of patients with that risk factor (not the full cohort). GCS: Glasgow Coma Scale; ICH: intracerebral hemorrhage; SAH: subarachnoid hemorrhage; AVM: arteriovenous malformation; IHD: ischemic heart disease; AF: atrial fibrillation; CKD: chronic kidney disease; COPD: chronic obstructive pulmonary disease

Characteristic	n (%)
Age, years	49.3±11.5¹
Sex	
Male	23 (58%)
Female	17 (43%)
Initial GCS	11.7±1.94
Pathology	
ICH	26 (65%)
Spontaneous SAH	10 (25%)
AVM	4 (10%)
Comorbidities	
Hypertension	21 (53%)
Diabetes mellitus	8 (20%)
Cardiac risk factors (n=11; 28%)	
Atrial fibrillation	4 (36%)
Heart failure	2 (18%)
IHD	3 (27%)
IHD+valvular AF	2 (18%)
Renal risk factors (n=4; 10%)	
Renal artery aneurysm	1 (25%)
CKD	2 (50%)
Renal artery stenosis	1 (25%)
Respiratory risk factors (n=3; 7.5%)	
COPD	2 (67%)
Aspiration pneumonia	1 (33%)
Graeb score	
Severe (9-12)	8 (20%)
Moderate (5-8)	19 (48%)
Mild (1-4)	13 (33%)
Hemiparesis	
Present	29 (73%)
Absent	11 (28%)

Group allocation and baseline between-group comparisons

The demographic and clinical characteristics are summarized in Table 2. There were no significant differences in age (median age of conservative 49 years; median age for surgery 48 years; U=194; P=0.871) and sex between the two groups (45% female in the conservative group versus 40% in the surgical group; OR=0.815, covered by a 95% confidence interval of 0.232 to 2.86; P=1.0). Both groups had similar distributions of Graeb severity categories for intracranial hemorrhage (Graeb severity categories measure the burden of IVH), with no significant difference (P=0.839), and non-significant trends in clot regression were seen on follow-up CT (75% of the conservative group compared to 55% of the surgical group showed evidence of clot regression by procalcitonin (PCT) analysis; P=0.282). Both groups had similar prevalence of hemiparesis at presentation, with 70% for the conservative group and 75% for the surgical group (OR=0.778 with a 95% CI from 0.193 to 3.13; P=1.0). There were no significant between-group differences with respect to the underlying causes of IVH (ICH, SAH, or AVM), as shown by a p-value of 0.568.

**Table 2:** Baseline characteristics and clinical variables of the conservative vs. surgical groups ^1^U=Mann-Whitney U test. ²Fisher's exact test. IQR: interquartile range; GCS: Glasgow Coma Scale; OR: odds ratio; CI: confidence interval; CT: computed tomography; ICH: intracerebral hemorrhage; sSAH: spontaneous subarachnoid hemorrhage; AVM: arteriovenous malformation

Variable	Conservative group (n=20)	Surgical group (n=20)	Test statistic
Age (median, IQR)	49 (11.8)	48 (14.3)	U=194; P=0.87^1^
Sex			
Female	45% (9/20)	40% (8/20)	Fisher's exact test; OR=0.815; 95% CI (0.232-2.86); P=1.0²
Male	55% (11/20)	60% (12/20)	
GCS	12.8±1.6	10.4±1.5	U=52.5; P<0.01^1^
Graeb score			
Severe	15% (3/20)	25% (5/20)	Fisher's exact test; P=0.839²
Moderate	50% (10/20)	45% (9/20)	
Mild	35% (7/20)	30% (6/20)	
Follow-up CT scan			
Decrease	75% (15/20)	55% (11/20)	Fisher's exact test; P=0.282²
Increase	10% (2/20)	5% (1/20)	
Same	15% (3/20)	40% (8/20)	
Hemiparesis: present	70% (14/20)	75% (15/20)	Fisher's exact test; OR=0.778; 95% CI (0.193-3.13); P=1.0²
Pathology			
ICH	65% (13/20)	65% (13/20)	Fisher's exact test; P=0.568²
sSAH	20% (4/20)	30% (6/20)	
AVM	15% (3/20)	5% (1/20)	
Hypertension: yes	60% (12/20)	45% (9/20)	Fisher's exact test; OR=0.545; 95% CI (0.155-1.91); P=0.527²
Diabetes: yes	30% (6/20)	10% (2/20)	Fisher's exact test; OR=0.259; 95% CI (0.0452-1.49); P=0.235²
Cardiac risk factor: yes	20% (4/20)	35% (7/20)	Fisher's exact test; OR=2.15; 95% CI (0.515​-​9.0)​​​;​​ P=0.48²
Renal risk factor: yes	15% (3/20)	5% (1/20)	Fisher's exact test; OR=0.298; 95% CI (0.283-3.15); P=0.605²

The comorbidity profile was similar for both groups: hypertension (60% in the conservative group; 45% in the surgical group; OR=0.545 with a 95% CI from 0.155 to 1.91; P=0.527), diabetes mellitus (30% in the conservative group; 10% in the surgical group; OR=0.259 with a 95% CI from 0.045 to 1.49; P=0.235), cardiac risk factors (20% in the conservative group; 35% in the surgical group; OR=2.15 with a 95% CI from 0.515 to 9.00; P=0.48), and renal risk factors (15% in the conservative group; 5% in the surgical group; OR=0.298 with an unspecified 95% CI; P=0.605).

An important baseline imbalance was observed with respect to neurological status; the surgical group had significantly lower admission GCS scores than the conservative group (GCS=10.4±1.5 vs. GCS=12.8±1.6; U=52.5; P=0.011), which reflects a greater degree of initial severity in the surgical group (Table 2).

Clinical outcomes by treatment arm

Apart from the factors in the baseline measurements, changes in functional symptoms as well as changes in imaging studies will yield critical data regarding how successful the treatment is.

The Course of Intraventricular Blood Burden

The patterns of blood clearance between treatment groups differed, as evidenced by the follow-up CT scan. For most patients under conservative management, there was a positive imaging outcome: 15 patients (75%) had a reduced intraventricular blood burden indicative of either spontaneous or medically induced clot regression, whereas three patients (15%) showed no change and another two patients (10%) had progressive hematomas. However, the imaging courses for patients under surgical management differed: 11 patients (55%) had a reduced blood burden, eight patients (40%) showed no change, and one patient (5%) had progressive hematomas. Although not statistically significant (P=0.20), these trends suggest a more stable imaging response among patients under conservative management (Table 3).

**Table 3:** Clinical outcomes between the conservative and surgical groups ¹Kruskal-Wallis test. ²Pearson's chi-squared test.

Variable	Conservative group (n=20)	Surgical group (n=20)	Test statistic
Follow-up CT scan			
Decrease	75% (15/20)	55% (11/20)	χ²^₂^=3.22; P=0.20²
Increase	10% (2/20)	5% (1/20)	
Same	15% (3/20)	40% (8/20)	
Improved state: yes	90% (18/20)	60% (12/20)	χ²^₁^=4.80; P=0.03²
Death: yes	10% (2/20)	40% (8/20)	χ²^₁^=4.80; P=0.03²

Factors associated with in-hospital mortality

The most important clinical outcome, survival versus mortality, showed clear differences between treatment groups and highlighted several consistent risk factors across the entire group.

Overall Mortality and Treatment-Arm Differences

Overall in-hospital mortality rate was 10 (25%). However, the surgical group had a significantly higher mortality rate at eight (40%) compared to the conservative group at two (10%; P=0.03). This fourfold difference in mortality between treatment groups stands out as the most striking finding in the data set. Notably, this difference occurred even though patients who underwent surgery had lower GCS scores at baseline, which indicated greater impairment and a worse prognosis. The unexpected outcome, where more impaired surgical patients had higher mortality than less impaired conservative patients, suggests that the type of treatment significantly impacted survival.

Univariate Predictors of Mortality in the Entire Cohort

To find the patient and disease characteristics linked to mortality, we compared survivors (n=30; 75%) and non-survivors (n=10; 25%) based on all baseline and imaging factors.

Demographic and Neurological Severity Markers

Non-survivors were significantly older, with an average age of 60.0±5.4 years, compared to survivors at 48.5±5.0 years (P=0.05). Even more striking, non-survivors had much lower GCS scores upon admission (mean 9.6±1.5) compared to survivors (mean 12.3±1.6; P<0.001). This indicates that severe neurological impairment at the time of presentation was a strong predictor of death. These findings show that age and acute neurological dysfunction consistently predict mortality.

Hemorrhage Burden Severity

The Graeb score category had the strongest association with mortality (P<0.001). Among non-survivors, seven (70%) had severe intraventricular bleeding (Graeb score 9-12), while moderate and mild cases accounted for one (10%) and two (20%), respectively. In contrast, survivors showed the opposite distribution: mild cases in 11 (36.7%), moderate in 18 (60%), and severe in only one (3.3%). This threefold difference in the prevalence of severe disease between deceased and surviving patients is the most powerful categorical predictor of mortality in this group.

Imaging Response and Prognosis

Follow-up CT imaging patterns provided clear prognostic insights. Among survivors, 26 (86.7%) showed reduced ventricular blood burden on follow-up scans, none (0%) had worsening conditions, and four (13.3%) remained stable. In sharp contrast, none of the non-survivors (0/10; 0%) showed any reduction in blood burden; instead, three (30%) had worsening hemorrhage and seven (70%) remained stable (P<0.001). This difference in imaging response is significant because it suggests that patients whose hemorrhage resolved or improved with treatment survived, while those with stable or worsening conditions all died. This imaging pattern might indicate a failure to clear blood from the subarachnoid or ventricular spaces or reflect inadequate initial hemorrhage management.

Comorbidity and Risk Factor Analysis

Cardiac risk factors were close to being significant as a mortality predictor, present in five (50%) of non-survivors compared to six (20%) of survivors (P=0.07). This trend implies that existing cardiac issues may reduce physiological resilience during acute intracranial hemorrhage. Other comorbidities, hypertension, diabetes, and kidney problems, did not show significant differences between survivors and non-survivors (all P>0.05), indicating that these chronic conditions did not independently predict acute mortality in this group.

Pathology Subtype and Motor Deficits

ICH (the main pathology subtype) was more common in non-survivors at nine (90%) compared to 17 (56.7%) of survivors, coming close to but not reaching statistical significance (P=0.15). The presence of hemiparesis (found in 70% of non-survivors and 73.3% of survivors; P=0.84) did not show any predictive value, suggesting that having motor deficits alone does not predict mortality independent of other factors (Table 4).

**Table 4:** Baseline characteristics and clinical factors by mortality (no vs. yes) ¹One-way ANOVA. ²Pearson's chi-squared test. CT: computed tomography; SD: standard deviation; ICH: intracerebral hemorrhage; sSAH: spontaneous subarachnoid hemorrhage; AVM: arteriovenous malformation; ANOVA: analysis of variance

Variable	No death (n=30)	Death (n=10)	Test statistic; p-value
Age (years), mean±SD	48.5±5.0	60.0±5.4	F₁,₃₈=4.14; P=0.05¹
Sex			
Male	56.7% (17/30)	60% (6/10)	χ²^₁^=0.03; P=0.85²
Female	43.3% (13/30)	40% (4/10)	
GCS	12.3±1.6	9.6±1.5	F₁,₃₈=21.04; P<0.01^1^
Graeb score			
Severe	3.3% (1/30)	70% (7/10)	χ²^₂^=21.26; P<0.01²
Moderate	60% (18/30)	10% (1/10)	
Mild	36.7% (11/30)	20% (2/10)	
Follow-up CT scan			
Decrease	86.7% (26/30)	0% (0/10)	χ²^₂^=26.42; P<0.01²
Increase	0% (0/30)	30% (3/10)	
Same	13.3% (4/30)	70% (7/10)	
Hemiparesis			
Present	73.3% (22/30)	70% (7/10)	χ²^₁^=0.04; P=0.84²
Absent	26.7% (8/30)	30% (3/10)	
Pathology			
ICH	56.7% (17/30)	90% (9/10)	χ²^₂^=3.82; P=0.15²
sSAH	30% (9/30)	10% (1/10)	
AVM	13.3% (4/30)	0% (0/10)	
Hypertension: yes	53.3% (16/30)	50% (5/10)	χ²^₁^=0.03; P=0.85²
Diabetes: yes	26.7% (8/30)	0% (0/10)	χ²^₁^=3.33; P=0.07²
Cardiac risk factor: yes	20% (6/30)	50% (5/10)	χ²^₁^=3.39; P=0.07²
Renal risk factor: yes	6.7% (2/30)	20% (2/10)	χ²^₁^=1.48; P=0.22²

Surgical subgroup: survivors versus non-survivors

Patients receiving surgical EVD were treated in a specific surgical cohort to evaluate if there were any differences in mortality predictive factors based upon treatment modality.

Summary of Surgical Patient Outcomes

All 20 surgical patients (100%) who were managed surgically were included in this analysis. Of these patients, eight (40%) died while in the hospital, and 12 (60%) survived until they were discharged. Thus, the operative mortality rate in this cohort was 40%, which is much higher than the 10% mortality rate for patients in the conservative cohort.

Surgical Patients' Demographics

There were no significant statistical differences between demographics for the surgical patients who survived and those who died while in the hospital. The median age of the surgical survivor patients was 46.5 years (IQR 40-55), while the median age for surgical non-survivor patients was 54.0 years (IQR 43.8-65.8; P=0.19). The gender distribution was also similar between the two groups, with five (41.7%) of the surgical survivors being women and three (37.5%) of the surgical non-survivors being women (P=0.85). Therefore, demographic characteristics do not play a significant role in the surgical outcome results when looking at the surgical cohort alone.

Hemorrhage Severity Predicts Surgical Outcomes

The initial Graeb scores were identified as a strong predictor of surgical survival (P<0.01). All surgical survivors (0/12, 0%) presented with no severe IVH (Graeb score 9-12). Instead, eight (66.7%) of the surgical survivors had a moderate Graeb score, and four (33.3%) had a mild Graeb score. In contrast, among the surgical non-survivors, five (62.5%) had a Graeb score of 9-12, while one (12.5%) had a moderate and two (25%) had a mild Graeb score. Thus, the severity of hemorrhage at the time of hospitalization is a predictor of surgical mortality; this corresponds with the overall observations for this cohort.

Imaging Response of Patients Following Surgery

The patterns of follow-up CT scans were clearly different for those with a favorable (survivors) or unfavorable (non-survivors) outcome following surgery (P<0.01). In the surgical survivors, 11 of 12 (91.7%) achieved an improvement (decrease) in their ventricular blood volume on follow-up imaging studies, 0/12 achieved progression, and only 1/12 (8.3%) experienced no change on follow-up imaging studies. In contrast, none of the surgical non-survivors (0/8, 0%) were noted to have reduced blood volume on follow-up imaging studies, and 1/8 (12.5%) experienced no change on follow-up imaging studies, while 7/8 (87.5%) either progressed or showed no improvement. These divergent imaging responses suggest that those surgical patients who had evidence of resolution of their hemorrhage were more likely to survive than those who experienced no significant reduction in their ventricular blood volume after the insertion of an EVD.

Other Prognostic Indicators for Surgical Patients

Factors such as hemiparesis status, pathology type, and number of comorbidity factors (hypertension, diabetes, cardiac issues, and renal issues) did not demonstrate a statistically significant difference between those surgical patients who were survivors and those who were not (all P>0.05). Thus, it appears that motor deficit status and the overall burden of chronic disease on surgical patients did not have an independent effect on their outcomes; rather, the severity of the hemorrhage and the response to treatment were the main predictors of response to surgical intervention (Table 5).

**Table 5:** Comparison between the survived and death groups in the surgical group ¹One-way ANOVA. ²Pearson chi-squared test. ANOVA: analysis of variance; ICH: intracerebral hemorrhage; SAH: subarachnoid hemorrhage; AVM: arteriovenous malformation; CT: computed tomography

Variable	No (n=12)	Yes (n=8)	Test statistic; p-value
Age (years)	40.0 (46.5-55.0)	43.8 (54.0-65.8)	F_1,18_=1.81; P=0.19¹
Sex	5/12 (41.7%)	3/8 (37.5%)	χ²^₁^=0.03; P=0.85²
Graeb score			
Severe	0/12 (0%)	5/8 (62.5%)	χ²^_2_^=10.74; P<0.01²
Moderate	8/12 (66.7%)	1/8 (12.5%)	
Mild	4/12 (33.3%)	2/8 (25%)	
Follow-up CT scan			
Decrease	11/12 (91.7%)	0/8 (0%)	χ²^_2_^=16.35; P<0.01²
Increase	0/12 (0%)	1/8 (12.5%)	
Same	1/12 (8.3%)	7/8 (87.5%)	
Hemiparesis: absent	2/12 (16.7%)	3/8 (37.5%)	χ²^₁^=1.11; P=0.29²
Pathology			
ICH	6/12 (50%)	7/8 (87.5%)	χ²^_2_^=3.07; P=0.22²
SAH	5/12 (41.7%)	1/8 (12.5%)	
AVM	1/12 (8.3%)	0/8 (0%)	
Hypertension: yes	5/12 (41.7%)	4/8 (50%)	χ²^₁^=0.13; P=0.71²
Diabetes: yes	2/12 (16.7%)	0/8 (0%)	χ²^₁^=1.48; P=0.22²
Cardiac risk factor: yes	3/12 (25%)	4/8 (50%)	χ²^₁^=1.32; P=0.25²
Renal risk factor: yes	0/12 (0%)	1/8 (12.5%)	χ²^₁^=1.58; P=0.21²

Conservative subgroup: survivors versus non-survivors

Predictors of Mortality Agreement

All surgical and conservative cohorts agreed that the severity of the Graeb score, along with response imaging on follow-up neuroimaging parameters, were the strongest prognostic variables. Baseline severe hemorrhage and the failure of blood burden reduction on follow-up imaging predicted mortality in both arms. In contrast, comorbidity burden and motor deficit status in both arms failed to independently predict outcome. These agreements suggest that hemorrhage severity and physiologic responses to hemorrhage (as indicated by imaging evolution) are primary biologic determinants of survival that predate treatment modality.

Differential Mortality Rates According to Treatment Arm

Despite similar distributions of baseline hemorrhage severity (Graeb scores; P=0.73) and similar overall baseline disease burden (i.e., hypertension, diabetes, and cardiac/renal risk factors; all P>0.05), the mortality rate was fourfold higher in the surgical arm (40%) than with conservative management (10%; P=0.03). Convalescent outcomes were especially glaring, considering the surgically managed patients entered with lower baseline GCS scores, offering a poor prognostic outlook, so as to afford the superior course of conservative management. Such a differential presents the conclusion that surgical intervention involving EVD must not have improved outcomes and, in fact, contributed to the increased mortality as a consequence of possible complications (infection, catheter obstruction, hemorrhagic complications) that offset benefits from direct hemorrhage evacuation.

Imaging Evolution as a Major Determinant of Survival

In all analyses, the imaging patterns of patients who survived (or "survivors") consistently demonstrated the largest and clearest relationship with the patient's ultimate survival. All survivors exhibited a decrease in blood burden on imaging obtained during the follow-up period, while all non-survivors exhibited either an unchanged or increased blood burden. This information is valid for both the individual treatment arms and the entire cohort of study participants. It appears that the way that spontaneous intracranial hemorrhages resolve (whether surgically or medically) or worsen defines the main reason for whether a patient survives an episode of spontaneous intracranial hemorrhage (Table 6).

**Table 6:** Comparison between the survived and death groups in the conservative group ¹One-way ANOVA. ²Pearson chi-squared test. ANOVA: analysis of variance; CT: computed tomography; ICH: intracerebral hemorrhage; SAH: subarachnoid hemorrhage; AVM: arteriovenous malformation

Variable	Survived (n=18)	Death (n=2)	Test statistic; p-value
Age (years)	49.0±10.2	65.0±0.0	F_1,18_=5.79; P=0.03
Sex (female)	8 (44.4%)	1 (50%)	χ²^₁^=0.02; P=0.88
GCS	12.9±1.5	9.7±1.0	F_1,18_=5.70; P=0.03
Graeb score			
Severe	1 (5.6%)	2 (100%)	χ²^_2_^=12.59; P<0.01
Moderate	10 (55.6%)	0 (0%)	
Mild	7 (38.9%)	0 (0%)	
Follow-up CT scan			
Decrease	15 (83.3%)	0 (0%)	χ²^_2_^=20.00; P<0.01
Increase	0 (0%)	2 (100%)	
Same	3 (16.7%)	0 (0%)	
Hemiparesis: absent	6 (33.3%)	0 (0%)	χ²^₁^=0.95; P=0.33
Pathology			
ICH	11 (61.1%)	2 (100%)	χ²^_2_^=1.20; P=0.55
SAH	4 (22.2%)	0 (0%)	
AVM	3 (16.7%)	0 (0%)	
Hypertension: yes	11 (61.1%)	1 (50%)	χ²^₁^=0.09; P=0.76
Diabetes: yes	6 (33.3%)	0 (0%)	χ²^₁^=0.95; P=0.33
Cardiac risk factor: yes	3 (16.7%)	1 (50%)	χ²^₁^=1.25; P=0.26
Renal risk factor: yes	2 (11.1%)	1 (50%)	χ²^₁^=2.14; P=0.14

## Discussion

This study investigated the clinical outcomes and prognostic factors in patients with spontaneous IVH, comparing conservative and surgical management strategies. Our findings reveal several critical insights into the management and prognosis of IVH, particularly highlighting the differential outcomes associated with treatment approaches and the significant impact of baseline neurological status and IVH burden on patient survival.

Significance of baseline severity as a primary determinant of group assignment and prognosis

In this study, the surgical group had a significantly lower mean admission GCS (10.4±1.5) than the conservatively managed group (12.8±1.6; P<0.011). This suggests we assign sicker patients (based on level of consciousness) to more invasive management. Similar patterns have been demonstrated in large observational studies; patients with EVD were younger than those without EVD, but they also had larger IVH volumes and lower GCS [14]. Moreover, in another cohort study, GCS ≤8 and a Graeb score >5 were shown to be strong predictors of EVD placement [15]. The congruence of our data with that above further solidifies that baseline severity is not equally distributed between treatment arms and must be accounted for when interpreting differences in outcomes.

Comparative outcome: conservative vs. surgical treatment

Although traditional management paradigms favor EVD placement for acute hydrocephalus or large intraventricular clot burdens, our findings contradict the suggestion that surgical drainage is invariably superior. In our series, patients with moderate clinical severity (fair GCS >10, moderate Graeb scores) had improved functional recovery with conservative management (90% vs. 60%) and decreased in-hospital mortality (10% vs. 40%) compared to those treated surgically. These results are highly consistent with the Egyptian comparative cohort [16], where patients with preserved GCS and moderate IVH load experienced excellent outcomes with conservative treatment (~90% good outcome, ~10% mortality), whereas EVD insertion was linked with worse short-term outcomes.

This trend is consistent with worldwide evidence that EVD by itself might not be useful for survival unless accompanied by effective clot removal or hydrocephalus drainage. In the CLEAR III randomized controlled trial, Hanley et al. demonstrated that stand-alone standard EVD did not lead to improved functional outcomes compared with conservative management, but that adjunctive intraventricular alteplase hastened clot resolution and reduced mortality. Similarly, further analyses also indicated that partial clot removal without a sufficient decrease in both parenchymal and intraventricular volume does not lead to neurological improvement [8,17].

Several meta-analyses have corroborated these findings, that EVD placement itself aids predominantly those patients who have obstructive hydrocephalus or deteriorating consciousness, but may have no effect, or even be harmful, if placed prophylactically in poor GCS patients [18]. Procedure-related infection, catheter blockage, and secondary hemorrhage remain major complications, particularly in resource-scarce settings. Collectively, these data show that conservative management is similarly efficacious and safer in patients with moderate IVH severity and stable neurological status, reserving EVD for patients who present with worsening or hydrocephalic status [9,19].

Clot burden, imaging dynamics, and survival

Our cohort also revealed that the Graeb score severity remains a powerful prognostic marker; 70% of non-survivors presented with severe intraventricular clot load compared with only 3.3% of survivors (P<0.01). This is consistent with multiple large studies and meta-analyses showing that a higher Graeb score (typically ≥5) is strongly associated with worse functional and mortality outcomes after IVH; patients with lower Graeb scores have outcomes comparable to those without IVH extension.

In our series, all survivors had radiologic evidence of clot regression on follow-up imaging (86.7%), which was not seen in non-survivors, a finding duplicated by Bisson et al. [20] and Morgan et al. [2], demonstrating that persistent blood burden in the ventricles is associated with increased hydrocephalus, intracranial hypertension, and neurotoxicity from blood breakdown products. Recent suggestions by other studies also emphasize the importance of early clot resolution to allow neurological improvement and to prevent secondary brain injury [21,22].

Recent randomized controlled trials also highlight that the active removal of the clot, either endoscopic or pharmacologic (e.g., via fibrinolysis), can reverse these adverse effects. For example, a 2023 multicenter randomized controlled trial demonstrated that the combination of intraventricular fibrinolysis with endoscopic evacuation led to higher rates of clot clearance, lower shunt dependency, and improved functional outcomes relative to standard drainage. Meta-analytic evidence from studies also demonstrated that early rapid clot clearance, ≥60% volume reduction within 72 hours, was independently associated with better mRS scores and lower mortality [23,24].

These collective data strongly reinforce that the degree and timing of ventricular clot removal, rather than simply the act of drain insertion, determine patient outcomes. Thus, our observed advantage in the non-surgical group may reflect both less severe initial pathology and the avoidance of EVD-related complications without impeding spontaneous clot resorption.

Age and comorbidity interplay

Age and comorbidity interaction demonstrated significant prognostic relevance in our cohort, better defining the pivotal features of the pathophysiology and clinical evolution of IVH. Of significant interest, increased age was highly associated with mortality in univariate analysis (mean 60.0±5.4 years in non-survivors and 48.5±5.0 years in survivors; P=0.05). This finding agrees with extensive epidemiological and clinical data demonstrating age to be a robust independent predictor of mortality and poor functional outcome in all groups of hemorrhagic stroke, including IVH. Even though widespread common medical comorbid conditions such as hypertension, diabetes mellitus, cardiac risk factors, and renal impairment did not independently predict death when adjusted by neurological status and severity of hemorrhage in our sample, this is in agreement with prior adjusted analysis [25].

A recent critical review of external ventricular drain placement after ICH with IVH concluded that, after controlling for admission GCS, hematoma and ventricular size, and other clinical covariates, patient age was still among the strongest predictors of 90-day mortality. This reinforces the importance of physiological and potentially neurovascular vulnerability associated with age, which appears to add to the detrimental effect of hemorrhagic injury over radiological severity and comorbidity index [26].

Implications for treatment strategy and decision-making

These observations have significant implications for IVH therapeutic decision-making and treatment protocols. Our findings, in conjunction with future literature, refute the dogma of standardized surgical intervention (e.g., early EVD) in all forms of IVH. Instead, we advocate a tailored approach in relation to clinical severity scores. In patients with a favorable neurological status, having moderate GCS and moderate intraventricular blood burden, conservative management by medical means can lead to improved clinical outcomes with minimal procedural risk. However, those with low GCS persisting, high Graeb score indicating high intraventricular clot burden, and failure of spontaneous clot resorption on follow-up imaging necessitate the urgent escalation of treatment [23].

This augmentation could include advanced interventions such as combined endoscopic hematoma evacuation with intraventricular fibrinolytic therapy, methods that have already been shown to decrease the rate of complications such as shunt dependency and improve neurological recovery. Our findings reaffirm that the clinical value of EVD placement depends on achieving significant clot clearance and not just the insertion of a drain. Where baseline neurological impairment is profound, mere drainage without resolution of the clot will not necessarily improve survival, highlighting strategic selection and appropriate aggressive treatment where indicated [24].

These data favor a more considerate clinical practice promoting individually tailored protocols as a function of age, neurological status, radiological burden, and dynamic imaging follow-up, with the intention of optimizing outcome while minimizing inbuilt procedural risk.

Limitations and future directions

First, there are several major limitations to this study: its nonrandomized observational design introduces confounding by indication. Patients managed with EVD presented with worse baseline neurological status, reflected by significantly lower admission GCS scores, and likely had more severe hydrocephalus and greater IVH burden. Because no multivariable or propensity-based adjustment was performed, causal inference regarding treatment effects cannot be made, and observed differences should be interpreted as associations only.

Second, outcomes were limited to in-hospital measures assessed at discharge. No standardized long-term functional outcomes, such as 30- or 90-day mRS, GOS-Extended, quality of life, or shunt dependency, were collected; therefore, the findings reflect short-term neurological course rather than durable functional recovery.

Third, the burden of IVH and radiologic evolution of it were assessed in categories using Graeb severity rather than quantitative volumetric measurements. No blinded dual reading, assessment of inter-rater reliability, or objective hydrocephalus indices were performed to introduce possible measurement bias.

Fourth, the mixed retrospective-prospective design may have introduced information bias due to differences in the completeness of the documentation, timing of imaging, and clinical practice patterns across study phases. Internal consistency may be further limited by the possible temporal evolution of the care protocols.

Additional limitations include the lack of standardized conservative and EVD management protocols, the absence of time-dependent exposure modeling (raising the possibility of immortal time bias), unmeasured co-interventions, incomplete reporting of safety outcomes (including ventriculitis and permanent CSF diversion), small sample size combined with multiple exploratory comparisons, and limited generalizability due to the single-center setting.

## Conclusions

The prospective and retrospective analysis of a cohort of patients from one institution demonstrates that EVDs are associated with greater short-term discharge GCS and lower raw rates of death in the hospital compared to conservative treatment. On the other hand, the baseline neurological condition of patients who received EVDs was significantly poorer than that of those who received conservative therapy, making it difficult to determine if the two types of treatment have a direct causal relationship. As a result, the conclusions presented here serve only as a starting point for further research to be conducted in prospective studies that will use standardized imaging methods, include a long-term follow-up factor that is functional, and provide a complete evaluation of each method's safety.
